# Ola1p trafficking indicates an interaction network between mitochondria, lipid droplets, and stress granules in times of stress

**DOI:** 10.1016/j.jlr.2023.100473

**Published:** 2023-11-09

**Authors:** Melanie Kovacs, Florian Geltinger, Lukas Schartel, Simon Pöschl, Peter Briza, Manuel Paschinger, Kitti Boros, Thomas Klaus Felder, Herbert Wimmer, Jutta Duschl, Mark Rinnerthaler

**Affiliations:** 1 Department of Biosciences, Paris-Lodron University Salzburg, Salzburg, Austria; 2 Institute of Functional Anatomy, Charité – Universitätsmedizin Berlin, Corporate Member of Freie Universität Berlin and Humboldt-Universität zu Berlin, Berlin, Germany; 3 Biocentre, Departments of Biology and Chemistry, Johannes Gutenberg University and Institute of Molecular Biology, Mainz, Germany; 4 Department of Laboratory Medicine, Paracelsus Medical University, Salzburg, Austria

**Keywords:** protein detoxification, protein aggregation, protein shuttling, stress response, lipid droplets, mitochondria, triacylglycerol, apoptosis, lipids/oxidation

## Abstract

Protein aggregates arise naturally under normal physiological conditions, but their formation is accelerated by age or stress-induced protein misfolding. When the stressful event dissolves, these aggregates are removed by mechanisms, such as aggrephagy, chaperone-mediated autophagy, refolding attempts, or the proteasome. It was recently shown that mitochondria in yeast cells may support these primarily cytosolic processes. Protein aggregates attach to mitochondria, and misfolded proteins are transported into the matrix and degraded by mitochondria-specific proteases. Using a proximity labeling method and colocalization with an established stress granule (SG) marker, we were able to show that these mitochondria-localized aggregates that harbor the “super aggregator” Ola1p are, in fact, SGs. Our in vivo and in vitro studies have revealed that Ola1p can be transferred from mitochondria to lipid droplets (LDs). This “mitochondria to LD” aggregate transfer dampens proteotoxic effects. The LD-based protein aggregate removal system gains importance when other proteolytic systems fail. Furthermore, we were able to show that the distribution of SGs is drastically altered in LD-deficient yeast cells, demonstrating that LDs play a role in the SG life cycle.

Lipid droplets (LD) are cellular organelles and composed of a neutral lipid-rich core that mainly consists of triacylglycerols and sterol esters (1). A protein-decorated phospholipid monolayer surrounds this core. This layer contains various proteins that allow LDs to interact with other organelles as well as synthesize and export fatty acids (2, 3). LDs also function as a storage space for phospholipids and proteins. In recent years, it has become obvious that LDs play a significant role in protein homeostasis, and this specific function is in the focus of the current study. Three different pathways are active in yeast cells, by which LDs remove misfolded and potentially toxic proteins.

1. Because of its oxidizing environment, the endoplasmic reticulum (ER) is prone to protein misfolding. These proteins are recognized by molecular chaperons, exported from the ER lumen, ubiquitinated and degraded by the 26 S proteasome (for detailed reviews, see Refs. (4, 5). Recently, it was shown that LDs originate at ER resident chaperone sites, take up certain polyubiquitinated proteins, move to the vacuole, and are taken up by the vacuole in a process known as microlipophagy (6).
2. Furthermore, LDs interact physically with cytosolic inclusion bodies. These LDs are a well-defined LD subset characterized by Pdr16p expression (7). When these LDs come into contact with inclusion bodies, they start to secrete sterols, which function as a detergent, aiding in the dissolving of these aggregates (8).
3. LDs are excellent stress, apoptosis, and aging sensors (9, 10, 11). These specific cellular conditions result in a massive increase in LD amounts. Furthermore, mitochondrial interaction is increased, and some proteins (e.g., misfolded proteins; proapoptotic and antiapoptotic proteins) that accumulate on top of mitochondria are passed on to LDs (9, 10). As a result, various mitochondrial functions are preserved from damage, and cellular fitness is improved, resulting in an increased chronological and replicative life span (11).

It was additionally shown in higher eukaryotes that LDs can specifically interact with intact proteins. As a result, cellular functions including gene expression are regulated (3). Nuclear LDs in *Drosophila melanogaster* serve as a histone deposit in oocytes (12). In mammals, LDs can bind NFAT5 (nuclear factor of activated T cells 5) preventing this transcription factor from entering the nucleus. As a result, functions including osmoprotection and inflammation are affected (13). PLIN5-decorated LDs are either associated with mitochondria or can shuttle into the nucleus to interact with the transcriptional coactivator peroxisome proliferator-activated receptor gamma coactivator 1-α and the NAD^+^-dependent deacetylase sirtuin 1. These nuclear LDs can prevent mitochondrial dysfunction by altering cellular protein expression levels (14). Recently it was demonstrated for mammals that LDs show an association with a special kind of protein aggregates, namely stress granules (SGs). These are considered as membrane-less organelles involved in RNA storage and cytoprotection (15). An interplay of LDs and SGs in fact regulates the mitochondrial uptake of fatty acids via the voltage-dependent anion channel (16).

It has recently become obvious that mitochondria play a crucial role in cellular homeostasis. It was demonstrated that mitochondria can assist the cytosolic proteasomal machinery in the degradation of damaged proteins. Aggregates form in response to stress (e.g., heat stress) and can attach to the outer mitochondrial membrane. Some proteins of these aggregates are transported into the mitochondrial matrix for degradation by the matrix resident LON protease. This specific protein degradation mechanism was termed MAGIC (mitochondria as guardian in cytosol) (17). Previously, we examined changes of the proteome of mitochondria in response to replicative aging, stress, and apoptosis. The mitochondrial proteome changed under the conditions chosen, and more than 500 proteins shuttled to mitochondria. For LDs, a similar stress-induced protein accumulation was also observed. In total, 112 proteins relocalized to both organelles upon stress application (10). The presence of a V-shaped hairpin domain in several of these proteins appears to mediate a shuttling from mitochondria to LDs. This V-domain has several similarities to hairpin structures identified in LD-resident proteins (class I LD proteins) (3, 9). All our data suggest that mitochondria-LD protein shuttling may act as a detoxifying process that improves mitochondrial and cellular fitness (9). Therefore, we aimed to analyze if there was any overlap because both systems (MAGIC and LD detoxification) that directly influence the mitochondrial proteome.

## Materials and Methods

### Yeast strains

The *Saccharomyces cerevisiae* BY4741 strain background (MATa *his3Δ1 leu2Δ0 met15Δ0 ura3Δ0*) was used for all experiments. Cells were cultivated at 28°C in complex medium (YPD/YPGal [1% (w/v) yeast extract, 2% (w/v) peptone, and 2% (w/v) d-glucose/galactose]) or synthetic complete glucose/galactose medium (SC-glucose/galactose) (2% [w/v] d-glucose/galactose, 0.17% [w/v] yeast nitrogen base without amino acids, 0.5% ammonium sulphate, and 10 ml of complete dropout mixture [0.2% Arg, 0.1% His, 0.6% Ile, 0.6% Leu, 0.4% Lys, 0.1% Met, 0.6% Phe, 0.5% Thr, 0.4% Trp, 0.1% Ade, 0.4% Ura, 0.5% Tyr per liter]) under constant shaking. By adding 2% (w/v) agar, solid media were prepared. Selection for plasmids was ensured by leaving out the respective amino acid(s).

### Cloning experiments and chromosomal integration

For the creation of the vector YEplac181-GPD, the GPD promoter was PCR amplified from the vector p416GPD (18) using the primers 5′-CCT GCA GGT CGA CTC TAG AGA GTT TAT CAT TAT CAA TAC TCG C-3′ and 5′-ATT CGA GCT CGG TAC CCG GGG ATC CTT TTT TTT TTA TCC GTC GAA ACT AAG TTC-3′ and cloned into the BamHI linearized vector YEplac181 (19) using Gibson assembly.

Vector YEplac181-GPD-*PET10*-mCHERRY was created in the following way: mCHERRY was PCR amplified from vector pFM571 (20) using the primers 5′-GTC GAC CAA TAT GGT GAG CAA GGG CGA G-3′ and 5′-ATT CGA GCT CGG TAC CCG GGT TAC TTG TAC AGC TCG TCC ATG-3′, and PET10 was amplified from chromosomal DNA using the primers 5′-TCG ACG GAT AAA AAA AAA AGG ATC CAT GTC TGA ATC ATC TAT TTC TTC TTC TAA AC-3′and 5′-TGC TCA CCA TAT TGG TCG ACA CAG CCG C-3′. Both products were cloned into the BamHI linearized vector YEplac181-GPD using Gibson assembly at the same time.

The plasmid YEplac181-GPD-*LOA1*-RFP was created by PCR amplification of LOA1-RFP from strain BY4741 LOA1::RFP::KanMX4 (9) using the primers 5′-CTA TGA CCA TGA TTA CGC CAT ATG GAA AAG TAC ACC AAT TGG AGA G-3′ and 5′-AGT CGA CCT GCA GGC ATG CAT TCC TTA GGC GCC GGT GGA GTG-3′. The resulting PCR product was cloned into the HindIII linearized vector YEplac181 via Gibson assembly. This vector was PCR amplified using the primers 5′-ACA GCT ATG ACC ATG ATT ACG-3′ and 5′-TTC CTG TGT GAA ATT GTT ATC C-3′. The GPD promoter was amplified with the primers 5′-ATA ACA ATT TCA CAC AGG AAA GTT TAT CAT TAT CAA TAC TCG CC-3′ and 5′-GTA ATC ATG GTC ATA GCT GTA TCC GTC GAA ACT AAG TTC TG-3′ from the vector p416GPD. Both PCR products were fused using Gibson assembly.

For the creation of YEplac181-GPD-*OLA1*-mCHERRY, the vector pUG35-*OLA1* was PCR amplified using the primers 5′-TCG ATA CCG TCG ACC TCG ACA TGG TGA GCA AGG GCG AG-3′ and 5′-TAA TTA CAT GAC TCG ACC AGT TACT TGT ACA GCT CGT CCA TG-3′. mCHERRY was amplified from vector pFM571 using the primers 5′-TCG ATA CCG TCG ACC TCG ACA TGG TGA GCA AGG GCG AG-3′ and 5′-TAA TTA CAT GAC TCG ACC AGT TAC TTG TAC AGC TCG TCC ATG-3′. Both PCR products were joint using Gibson assembly.

Plasmid pUG35-*OLA1* was created by PCR amplification of *OLA1* from chromosomal DNA using the primers 5′-TCC ATA CTC TAG AAC TAG TGG ATC CAT GCC TCC AAA GAA GCA AG-3′ and 5′-TAT CGA TAA GCT TGA TAT CGA ATT CAT TCT TAC CAG CAC CAG C-3′. The resulting product was cloned into the BamHI and EcoRI linearized vector pUG35 using Gibson assembly.

For creation of a chromosomal Ola1-GFP fusion, an integration cassette was created. GFP was amplified from the vector pUG35 using the primers 5′-CCG GAAT TCA TGT CTA AAG GTG AAG AAT T-3′ and 5′-CGC GGAT CCA GCG TCA AAA CTA G-3′. The resulting PCR product was cloned into the vector pUS19.12 (unpublished data; a pT//T3α-19 derivate) using the enzymes EcoRI and BamHI. The hygromycin resistance gene (hph) was amplified from an hph gene containing vector (21) using the primers 5′-CGC GGA TCC AGC GTC AAA ACT AG-3′ and 5′-ACG CGT CGA CCT GCA GAG GTA AAC CCA GAA-3′. The insert was cloned into the pUS19-GFP vector using BamHI and SalI, thus creating a hygromycin B-selectable GFP integration system. The integration cassette was amplified using the primers 5′-TCG TTG AAG ACG GTG ATA TCA TTT ACT TCA GAG CTG GTG CTG GTA AGA ATG GTT CTT CAG GAAG TTC GAT GTC TAA AGG TGA AGA ATT-3′ and 5′-ACA CAC ATA CAT AAA ATC CGA TGC CAT CCA CTC GCG AGA TTG CTT TTT TCC TGC AGA GGT AAA CCC AGA A-3′. An integrative transformation yielded a C-terminal GFP-tagged chromosomal version of *OLA1* that was positively selected by growth on YPD plates containing 300 mg/l hygromycin B and confirmed by PCR as well as sequencing.

### Yeast transformation

Yeast cells were grown to an absorbance of of 0.6–0.8 at 600 nm prior to harvesting. After centrifugation at 3,500 *g* for 3 min, cells were washed with LiAc/TE (100 mM Tris, 10 mM Tris, 1 mM EDTA, pH 8.0) and resuspended in 200 μl LiAc/TE. About 50 μl of this cell suspension were mixed with 5 μg plasmid DNA (plasmid preparation was done using the Promega PureYield™ Plasmid Miniprep System), 10 μg/ml single-stranded salmon sperm DNA, and 300 μl LPT (100 mM LiAc, 50% PEG 3350, 10 mM Tris, 1 mM EDTA, pH 8.0). Incubation at 28°C for 30 min under constant shaking was performed afterward. About 40 μl of DMSO was added, and the cells were heat-shocked for 15 min at 42°C. After regeneration in complex medium, cells were pelleted, resuspended in a small volume, and plated on the respective selective media.

### Mitochondria isolation

Cells were cultured in synthetic media (200 ml) overnight. After centrifugation for 10 min at 3,500 rpm, the pellets were resuspended in 2 ml ice-cold sorbitol B buffer (0.7 M sorbitol, 50 mM Tris [pH 7.5], and 0.2 mM EDTA). The cell suspension was mixed with glass beads (0.25–0.5 mm; one-third of volume). The samples were then homogenized for 4 × 10 s at 4.5 m/s in a bead beater homogenizer (FastPrep-24). The following centrifugation steps were all carried out at 4°C. Via centrifugation for 5 min at 800 *g*, the glass beads were pelleted, and the supernatants were transferred. A centrifugation step for 15 min at 15,000 *g* yielded a mitochondrial pellet, and the supernatant was discarded. The pellet was resuspended in 2 ml ice-cold sorbitol B buffer. After a centrifugation step at 800 *g* for 5 min, the supernatant was transferred into a new reaction tube. The probe was centrifuged again at 15,000 *g* for 15 min, and the cleaned mitochondrial pellet was resuspended in 1.5 ml sorbitol B buffer. The supernatant was transferred into a new reaction tube after centrifugation at 800 *g*. After the final centrifugation step (15,000 *g* for 15 min), the pellet was resuspended in 200 μl sorbitol B. The mitochondrial concentration (absorbance at _600 nm_) was measured using a NanoDrop™.

### LD isolation

An overnight culture (200 ml) was centrifuged for 3 min at 3,500 rpm, and the pellet was resuspended in Tris isolation buffer (0.1 M Tris-HCl [pH 7.4], 1 mM EDTA, 1 mM EGTA, 100 mM NaF, 1 mM PMSF, and 10 U/ml aprotinin). The cell suspension was supplemented with glass beads (one-third of the volume), and the cells were broken in a bead beater (4 × 10 s at 4 m/s). The beads were pelleted for 5 min at 4,000 rpm, and the supernatant was transferred into ultracentrifugation tubes. The samples were ultracentrifuged for 30 min at 100,000 *g*. Subsequently, the LDs floating on top were collected. To get rid of cytosolic contaminations, the samples were mixed with sucrose and sodium carbonate (final concentrations of 25% and 10 mM, respectively) and layered on top of a 1 ml 60% sucrose solution. The sample was then overlaid with 10 mM sodium carbonate followed by 1 ml Tris isolation buffer. After ultracentrifugation at 100,000 *g* for 30 min, the purified LDs were collected. The LD concentration (absorbance at 600 nm) was determined using a NanoDrop™.

### Shuttling

Isolated mitochondria as well as LDs were normalized to an absorbance of 0.2 at 600 nm. LDs were diluted in sorbitol B, and mitochondria were diluted in Tris isolation buffer. Afterward, varying LD amounts were added to a constant amount of mitochondria (mitochondria-LD ratio: 1:1, 1:2, 1:5, and 1:9; in samples, 1:1, 1:2, and 1:5 sorbitol B was added to achieve a total volume of 200 μl per sample).

The samples were then incubated at 28°C for 30 min under constant shaking (500 rpm). LDs and mitochondria were separated by a centrifugation step at 15,000 *g* for 15 min at 28°C. The supernatants, representing LDs, and the pellets (resuspended in 200 μl sorbitol B), representing mitochondria, were pipetted separately into a 96-well plate, control samples containing only LDs, mitochondria, and buffer were also added. In a final step, the GFP fluorescence was measured using a fluorometer.

### GFP fluorometric measurements

GFP fluorescence measurements were performed using the Anthos Zenyth 3100 (Anthos Labtec Instruments GmbH, Salzburg, Austria) (excitation: 535 nm; emission: 625 nm; detection time: 4 s). To compensate for clonal differences in GFP expression levels, stressed samples in each single case were normalized to the respective unstressed samples.

### Fluorescence microscopy

Microscopical analysis was performed with a Nikon (Tokyo, Japan) Eclipse Ni-U equipped with a DS-Fi2 digital camera, a Nikon Eclipse Ti2-E (Tokyo, Japan), and a Leica DMi8 microscope (Wetzlar, Germany) with an Abberior STEDYCON instrument (Abberior, Göttingen, Germany). Colocalization of two fluorescent proteins was quantified using the imaging software NIS-Elements (Nikon, Tokyo, Japan). This software determines the Pearson's correlation coefficient (PCC) and Mander's correlation coefficient (MCC). MCC ranges from 0 to 1, and PCC ranges from −1 to +1. In case of PCC, a positive value indicates a colocalization event, whereas a negative value indicates no colocalization. The higher the PCC and MCC, the better the colocalization. The PCC as well as MCC was calculated for 10 cells. Scale bars in all images represent 5 μm.

### Western blot and Coomassie staining

Samples (either isolated mitochondria or LDs; normalized to absorbance at 600 nm) were mixed with a SDS loading buffer, boiled for 5 min at 95°C, and separated on 15% SDS-PAGE gels. Afterward, the proteins were blotted on a BA85 nitrocellulose membrane (Schleicher & Schuell BioScience GmbH, Dassel, Germany) for 90 min with 250 mA. After blockage of the membrane with MTBS-T (25 mM Tris [pH 7.6], 137 mM NaCl; 0.1% Tween-20, 5% nonfat milk powder) for 90 min at room temperature, the primary anti-GFP antibody (B-2) (sc-9996 HRP; Santa Cruz) was added after a 1:1,000 dilution in TBS-T and was incubated overnight at 4°C on a shaker. The blot was then washed three times for 10 min with TBS-T at room temperature and was incubated with the secondary polyclonal rabbit anti-mouse immunoglobulins/HRP (P0161, Dako, 1:2,000 dilution with MTBS-T) for 2 h at room temperature under constant shaking. The membrane was washed once again three times with TBS-T and was developed in 2 ml SuperSignal™ West Dura according to the manufacturer's specifications (Thermo Fisher Scientific, Rockford, IL).

### Creation of BY4741 *OLA1*-TurboID strains

A TurboID-3xMYC-kanMX6 integration cassette was amplified from the vector pFA6a-TurboID-3MYC-kanMX6 (22) via PCR using the primers 5′-GTC GTT GAA GAC GGT GAT ATC ATT TAC TTC AGA GCT GGT GCT GGT AAG AAT CGG ATC CCC GGG TTAA TTA A-3′ and 5′-TAC ATA CAC ACA TAC ATA AAA TCC GAT GCC ATC CAC TCG CGA GAT TGC TTG AAT TCG AGC TCG TTT AAA C-3′. Upon integrative transformation, *OLA1* was tagged C-terminally with the ORF of the biotin ligase TurboID via homologous recombination. Positive clones were identified by growth on YPD plates containing 200 mg/l G418. Correct integration was confirmed by PCR and sequencing. For the creation of the pUG35 *OLA1*-TurboID-eGFP construct, chromosomal DNA from BY4741 *OLA1*-TurboID-3xMyc-kanMX6 was isolated. *OLA1*-TurboID was PCR amplified using the primers 5′-CAT ACT CTA GAA CTA GTG GAT CCA TGC CTC CAA GAA GAA GCA AG-3′ and 5′-TCC TGC AGC CCG GGA ACA GAT CTT CCT CAG AGA TGA G-3′ and cloned into the BamHI linearized vector pUG35 via Gibson assembly, thus rendering a C-terminal GFP fusion.

### Streptavidin bead pulldown

Yeast cells were grown overnight and diluted to an absorbance of 0.1 at 600 nm. After 18–24 h, growth in YPD or SC-biotin media, cells were pelleted and resuspended in 50 ml YPD. Biotin was added to achieve a final concentration of 50 μM. Cells were then stressed for a defined time (either 10 min or 20 min) at 46°C. After this heat shock, cells were centrifuged (3,500 rpm for 5 min at 4°C) and washed twice with dH_2_O. Pellets were lysed in RIPA buffer using glass beads. The bead beater was set to 5 m/s, and cells were shaken six times for 30 s with a 30 s rest on ice in between. The cell lysate was cleared by a centrifugation step (800 rpm for 5 min at 4°C). The supernatant was carefully transferred and recentrifuged for 10 min at 14,000 rpm. Protein concentration was measured using a BCA assay (Thermo Fisher Scientific, Rockford, IL). For the pulldown assay, 300 μl streptavidin magnetic beads (NEB, Ipswich, MA) were washed twice with RIPA buffer and then incubated with 6 mg protein at 4°C on a rotary shaker overnight. Beads were then washed two times with RIPA buffer, one time with 1 M KCl, one time with 0.1 M Na_2_CO_3_, one time with 2 M urea, and two times with Tris-HCl (pH 8.0). Beads were stored in 1 ml Tris-HCl (pH 8.0) at −20°C until further analysis.

### Mass spectrometry-based proteomics

Washed beads were suspended in 100 μl extraction buffer 2 (from the ProteoExtract All-in-One Trypsin Digestion Kit; Merck Millipore, Billerica, MA) and 100 μl of 100 mM ammonium bicarbonate. Disulfide bridges were reduced with DTT for 20 min at 60°C and alkylated with iodoacetamide for 1 h at room temperature. After the digest with trypsin (Thermo Scientific, Rockford, IL) for 12 h at 37°C, detergents present in the extraction buffer were precipitated by adding 500 μl of 0.5% trifluoroacetic acid. After centrifugation, the clear supernatant was desalted using 100 μl Pierce C18 Tips (Thermo Fisher Scientific). Peptides were separated by reverse-phase nano-HPLC (Dionex Ultimate 3000; ThermoFisher Scientific, Bremen, Germany), directly coupled via nano electrospray to a Q Exactive Orbitrap mass spectrometer (Thermo Fisher Scientific). The column (AcclaimPepMap RSLC C18, 75 μm × 15 cm; Dionex, Thermo Fisher Scientific) was developed with an acetonitrile gradient (solvent A: 0.1% [v/v] formic acid; solvent B: 0.1% [v/v] formic acid/90% [v/v] acetonitrile; 5–45% B in 120 min) at a flow rate of 300 nl/min at 55°C. Capillary voltage of the nano spray was 2 kV. Lock mass calibration was used for highest accuracy. Peptide fragmentation/identification was done with a top 12 method and a normalized fragmentation energy at 27%. Protein identification was done with PEAKS Studio X+ (Bioinformatics Solutions, Waterloo, Canada), using the yeast subset of the UniProt database. Settings for the searches: digest tool trypsin semispecific, −10lgP ≥35 (corresponding approximately to 0.1% false discovery rate). Fixed modification: carbamidomethylation (C), variable modifications: deamidation (NQ), oxidation (M). The mass spectrometry proteomics data have been deposited to the ProteomeXchange Consortium via the PRIDE (23) partner repository with the dataset identifier PXD045513 and 10.6019/PXD045513.

### Statistical analysis

The statistical significance of the overlap between two groups of genes was calculated with an online available tool (http://nemates.org/MA/progs/overlap_stats.html) leading to the representation factor and its *P* value. A representation factor of >1 suggests that more genes appear in the overlap as expected by chance. Data are presented as arithmetic means ± SD. One-Way ANOVA with Tukey honest significant difference analysis was applied in Figs. 3B, 5A, B, 6A–C, and 7B a Student's *t*-test was performed in Fig. 5C–F. **Significance was indicated as follows: ∗*P* < 0.1; ∗∗*P* < 0.05; and ∗∗∗*P* < 0.01.**

## Results

### Overlap between MAGIC and LD detoxification

**Fig. 1:**
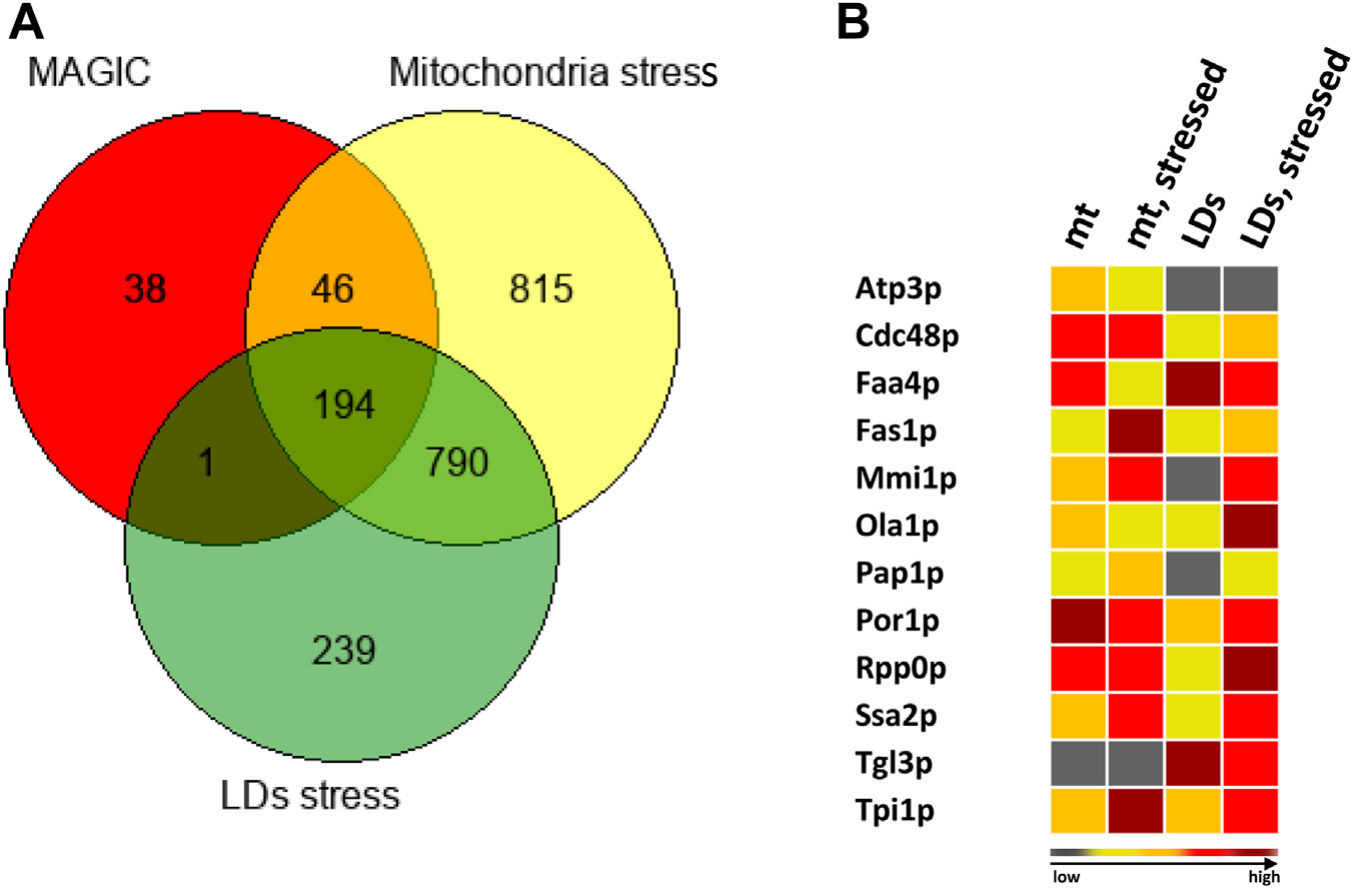
Overlap between MAGIC and LD detoxification. A: A Venn diagram is displayed that compares the obtained proteomes of LDs and mitochondria from stressed BY4741 cells (10) with a dataset of proteins that are subjected to mitochondrial protein degradation (MAGIC) (17). This figure shows the numbers of proteins who are affected by different forms of stress and degradation. B: Heat map (occurrence chart) of LD and mitochondrial protein localizations. With the exception of Tgl3p and Atp3, which are marker proteins for LDs and mitochondria, respectively, the displayed proteins were chosen from the results of the Venn diagram in (A). Gray indicates the absence or near absence of proteins. Different shades of red represent varying protein occurrence intensity amounts of proteins: yellow: low abundance; red: middle abundance; dark red: high abundance of proteins. Mt, mitochondria.

To compare whether MAGIC and LDs work synergistically to detoxify damaged proteins and protein aggregates, respectively, we used two existing datasets: *I*) potential MAGIC candidates (17) and *II*) mitochondrial and LD protein enrichment upon stress (10). In fact, a large overlap between MAGIC and LD detoxification was observed (Fig. 1A). We obtained representation factors (calculated with http://nemates.org/MA/progs/overlap_stats.html; *P* values <0.0001) >1, suggesting significance as follows: stressed LDs and MAGIC: 3.4; stressed mitochondria and MAGIC: 2.8; and stressed LDs and stressed mitochondria: 2.6. These results indicate a substantial overlap between proteins that are either removed by MAGIC or present at LDs. supplemental Table S1 summarizes 194 proteins identified by comparing the mitochondrial stress proteome, LD stress proteome, and MAGIC proteome. Figure 1B shows selected proteins from that overlap that, in most cases, exhibit the same properties: *1)* unstressed cells have a cytosolic distribution, *2)* stressed cells have a significant mitochondrial accumulation, and *3)* LD relocalization (3). Atp3p (part of the inner mitochondrial membrane) and Tgl3p (part of LDs) were chosen as marker proteins. Mmi1p may serve as a typical example in this heat map that supports our assumption. We previously showed that this cytosolic protein has a stress-dependent mitochondrial localization (24, 25). Mmi1p was later designated as a marker protein for the MAGIC pathway (17). Furthermore, we were able to show that Mmi1p shuttles to LDs in response to stress, showing a link between this protein’s LD and mitochondrial localization (9).

### The stress-dependent relocalization of Ola1p from mitochondria to LDs

**Fig. 2:**
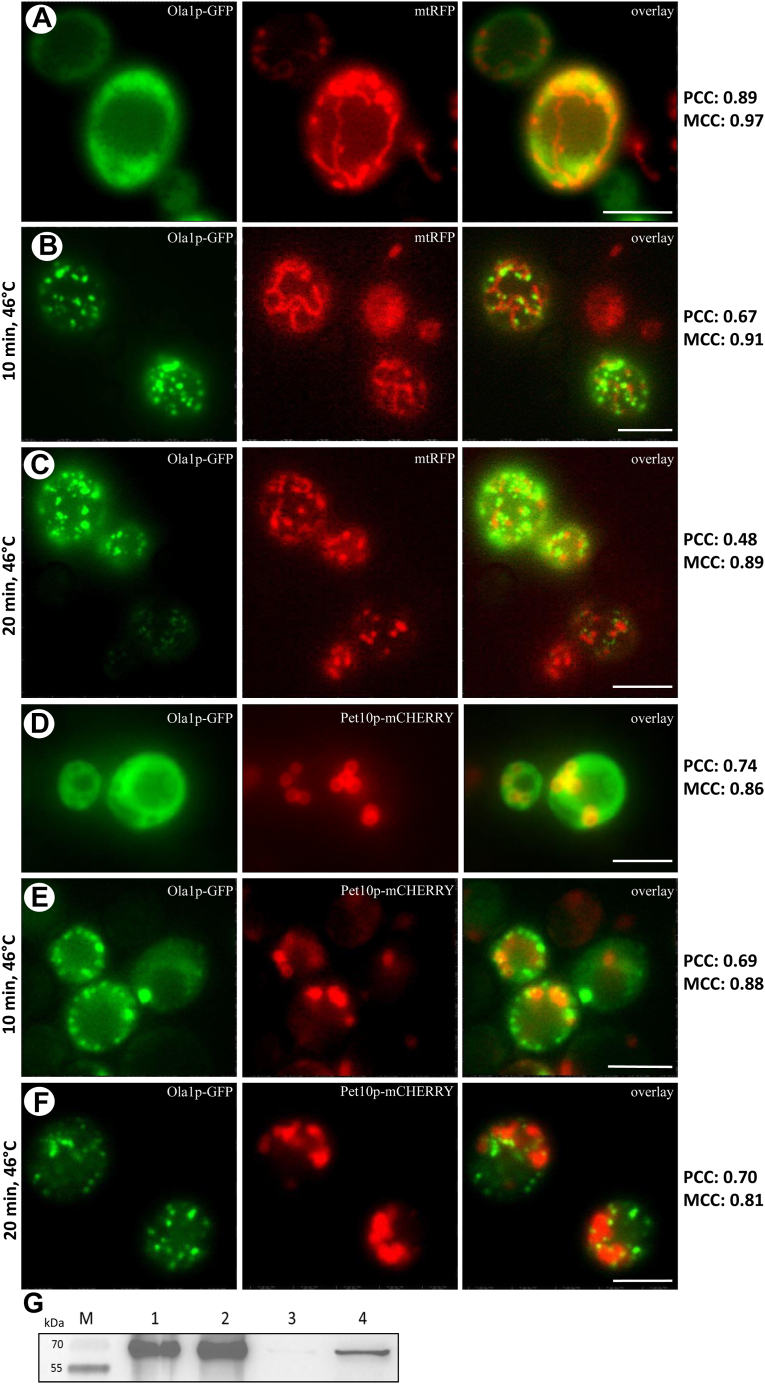
Localization of Ola1 upon stress and apoptosis. The strain BY4741 pUG35-*OLA1*-GFP pYX142-mtRFP was used in (A), (B), and (C), the strain BY4741 pUG35-*OLA1*-GFP YEplac181-GPD-*PET10*-mCHERRY in (D), (E), and (F), and the strain BY4741 pUG35-*OLA1*-GFP pCM666-mBAX in (F). A and D: 28°C Unstressed conditions are presented. B and E: Cells are shown after a 10 min heat stress (46°). C and F: Cells are shown after a 20 min heat stress (46°C). Scale bars represent 5 μm. G: A Western blot detecting Ola1-GFP (GFP-antibody [B-2] HRP [sc-9996 HRP; Santa Cruz]) either at mitochondria from nonapoptotic (1) and apoptotic (2) cells or LDs isolated from nonapoptotic cells (3) or apoptotic (4) cells is shown. Apoptosis was induced by expression of murine BAX after addition of 200 mg/l doxycycline. The associated loading control is presented in supplemental Fig. S1.

In a small screen performed on some of the identified proteins (Fig. 1B), Ola1 was found to be the most promising candidate, superior even to Mmi1p, and was therefore selected for further analysis. In this screen, protein aggregation and association with mitochondria were analyzed after stress application (heat stress; murine BAX-induced apoptosis, hydrogen peroxide treatment, and proteotoxic stress). This strong LD association of Ola1p upon stress is most probably attributed to the fact that this P-loop ATPase has already been labeled as “super aggregator” (26) and appears to have a role in protein homeostasis by interfering with translation (27) or proteasomal degradation (28). Ectopic expression of an Ola1p GFP fusion protein revealed a homogenous cytosolic distribution in either exponential growth phase or stationary growth phase (Fig. 2A, D). A 10 min heat stress resulted in complete redistribution and aggregation build-up of Ola1p. The distribution of these aggregates was further investigated using colocalization with the fluorescent mitochondrial marker mtRFP. Most “Ola1p aggregates” precisely align at the surface of mitochondria as a result of a 10 min heat stress (46°C), as seen in Fig. 2B. A shift in the location of these aggregates was observed after prolonged stress exposure (20 min, 46°C) (Fig. 2C). In fact, a drop in both the PCC and the MCC was calculated (both are coefficients quantifying colocalization (29)). Some of these aggregates interacted clearly with Pet10p-mCHERRY, a bona fide LD resident protein (30). Many aggregates surround LDs and, in some cases, sit on top of LDs (Figure 2E, F). Confocal microscopy (supplemental Movie S1) confirmed the tight association between some Ola1p granules and LDs.

**Supporting Figure S2:**
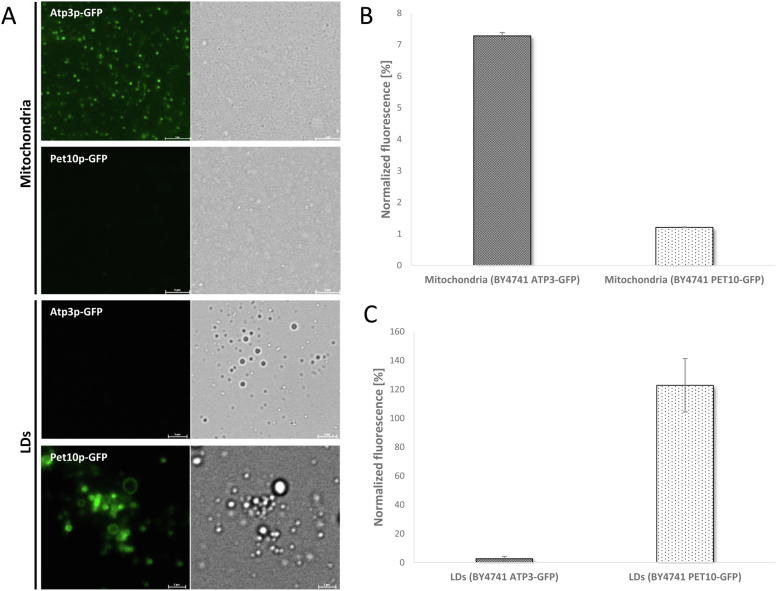
Purity control for isolated mitochondria and LDs. Mitochondria as well as LDs were isolated from the strains BY4741, BY4741 ATP3-GFP::HIS3MX6 and BY4741 PET10-GFP::HIS3MX6. Fluorescence of mitochondria and LDs was analyzed using fluorescence microscopy (A) and fluorometric measurements (B, C). A: Scale bars: 5 µm (Atp3-GFP and Pet10p-GFP mitochondria; Atp3-GFP LDs); 2 µm (Pet10p-GFP LDs). B: Fluorometric analysis of isolated mitochondria normalized to auto-fluorescence of mitochondria isolated from the strain BY4741. C: Fluorometric analysis of isolated LDs normalized to auto-fluorescence of LDs isolated from the strain BY4741.

Finally, Western blotting was used to determine whether Ola1p was present in mitochondria or LDs (Fig. 2G). Purity of mitochondrial and LD fractions was confirmed after isolation of LDs and mitochondria from strains either expressing Atp3p-GFP (mitochondrial marker protein) or Pet10p-GFP (LD marker protein) and their microscopic (supplemental Fig. S2A) and fluorometric analysis (supplemental Fig. S2B, C). Furthermore, we applied additional stressors because in a previous mass spectrometry-based work, we could demonstrate that multiple independent stressors result in changes to the mitochondria as well as LD proteome (10). Cell stress was induced by the ectopic expression of murine BAX. Apoptotic and nonapoptotic cells were broken with a bead beater, and mitochondria and LDs were isolated. An anti-GFP antibody was used to detect the presence of Ola1p-GFP at these two organelles. Ola1p was present in mitochondria of both apoptotic and nonapoptotic cells, as shown in Fig. 2G. In contrast to mitochondria, Ola1p was only present in LDs formed by apoptotic cells but absent in nonapoptotic LDs. These data suggest that Ola1p is shuttled to mitochondria and LDs in parallel, and/or that Ola1p is relocalized from mitochondria to LDs.

**Fig. 3:**
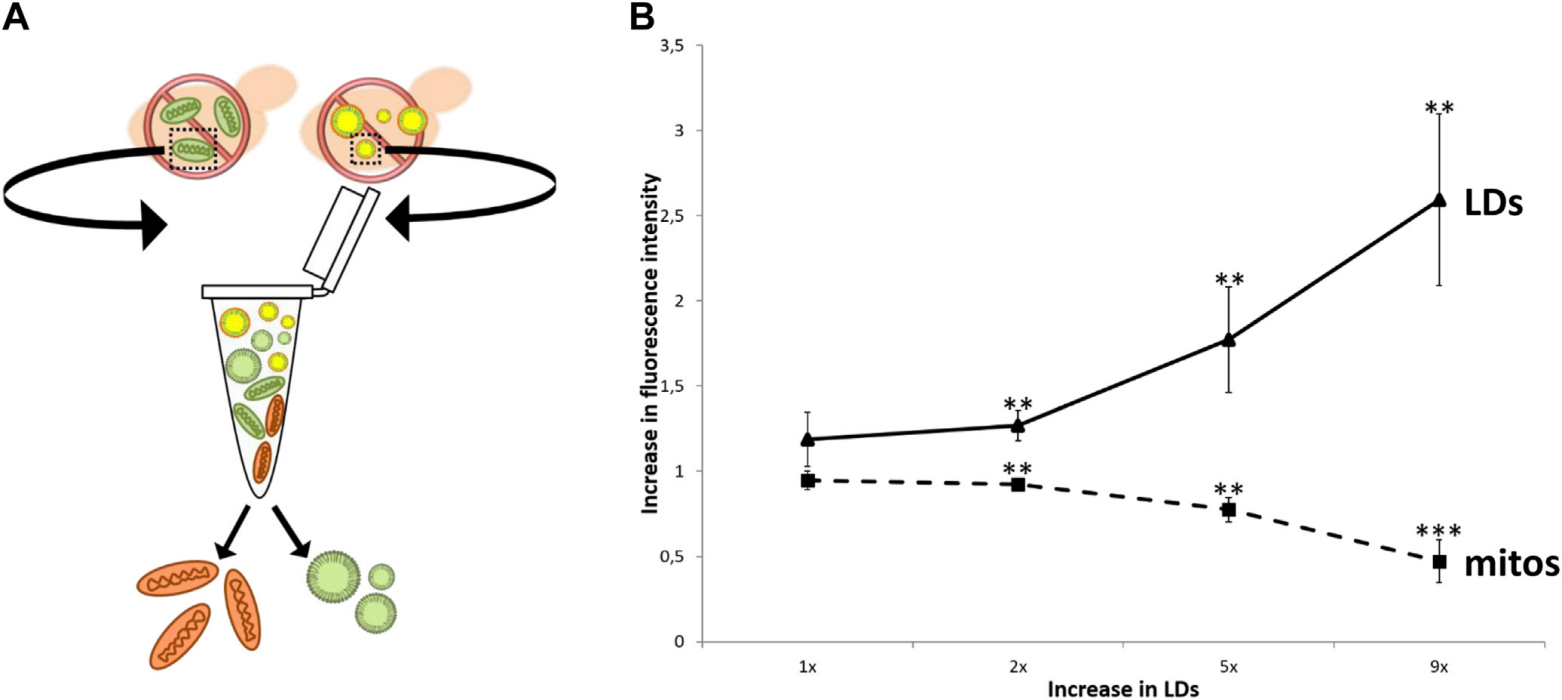
In vitro shuttling of Ola1p between mitochondria and LDs. A: Mitochondria containing Ola1p-GFP and nonlabeled LDs were isolated. Both organelles were mixed in a reaction tube and after 30 min of incubation separated again. The fluorescence of both organelles was either measured in a fluorimeter plate reader or detected by Western blot. B: The fluorescence of both organelles after combination and separation is shown. The dashed line represents mitochondria, and the continuous line represents LDs. The x-axis represents increasing amounts of LDs (e.g., 9× is a 9-fold excess of LDs).

As a result, we conducted an in vitro experiment to differentiate between the two options. Mitochondria were isolated from cells expressing Ola1-GFP ectopically, whereas LDs were acquired from control cells that did not express Ola1-GFP. As a result, mitochondria fluoresced brilliantly green, but LDs did not. Both organelles were then combined and incubated at 28°C before being reisolated (Fig. 3A).

**Supporting Figure S3:**
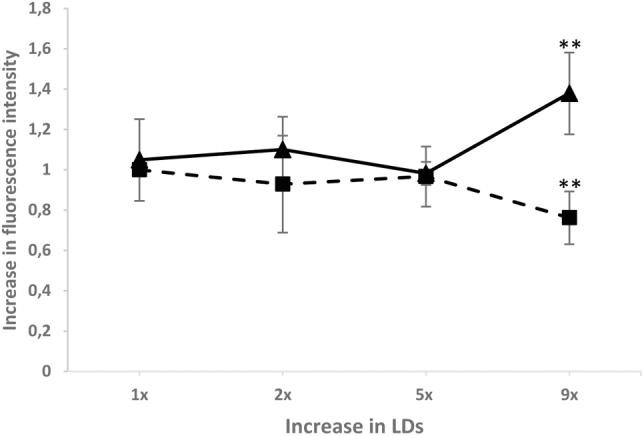
The fluorescence of ATP3-GFP at either mitochondria or LDs is shown after mixing and separation of both organelles. The dashed line represents mitochondria, the continuous line LDs. The *x*-axis represents increasing amounts of LDs (e.g., 9 x is a 9-fold excess of LDs).

When equal amounts (normalized to an absorbance at 600 nm) of LDs and mitochondria were combined, we observed a modest reduction of Ola1p-GFP at mitochondria, whereas the same protein started to appear at LDs. In contrast, a 9-fold increase in LDs resulted in almost total loss of Ola1p at mitochondria (Fig. 3B) and high levels of Ola1p at LDs, indicating that the number of LDs directly influences the process. In the absence of LDs, no protein loss was identified, ruling out passive Ola1p disappearance from mitochondria. Furthermore, a typical mitochondrial protein (Atp3; component of the F_0_F_1_ ATPase) showed no substantial shuttling from mitochondria to LDs in a control experiment (supplemental Fig. S3).

### Proximity labeling approach and identification of the stress-dependent Ola1p interactome

**Supporting Figure S4:**
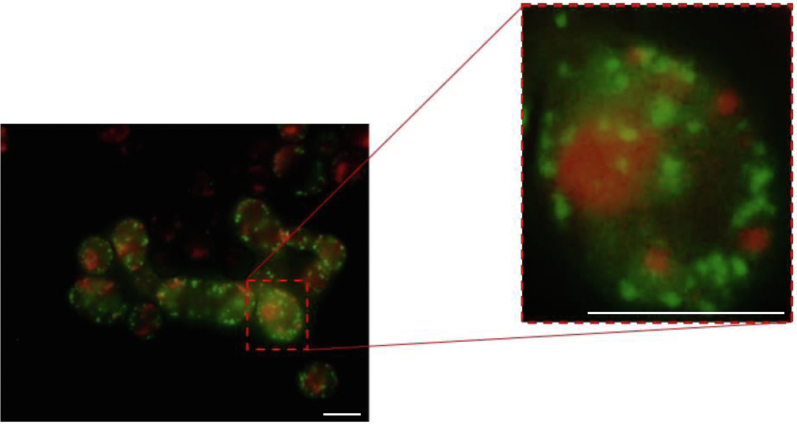
Fluorescence image of BY4741 pUG35-*OLA1*-TurboID-GFP YEplac181-GPD-PET10-mCHERRY cells upon heat stress. This image demonstrates that *1*) both Ola1p-GFP and Ola1p-TurboID-GFP do not differ in their localization and that *2*) Ola1p aggregates surround LDs or are in close proximity. Scale bars: 5 µm.

A proximity labeling approach was used to gain insight into the nature of protein aggregates that attach to either mitochondria or LDs and to identify putative Ola1p docking sites at both organelles. Therefore, we created a strain expressing an Ola1p-TurboID fusion, a protein fusion that contains a biotin ligase, which biotinylates all proteins in close proximity (31). An independent control experiment confirmed biotinylation in cell lysates by streptavidin blots (data not shown). Furthermore, ectopic expression of Ola1p-TurboID-GFP after a 20 min heat stress confirmed its localization to LDs. As a result, we conclude that the fusion protein preserves its original function and localization (supplemental Fig. S4). To identify biotinylated proteins, we incubated cell lysates from Ola1p-TurboID and control cells with streptavidin magnetic beads. Mass spectrometry was used to identify proteins in Ola1p-containing aggregates in enriched cell eluates from unstressed and heat-shocked stressed cells.

The stress-induced Ola1p interactome included 462 proteins when compared with unstressed cells (96 proteins were specific for a 10 min heat stress, and 157 proteins were specific for a 20 min heat stress) (supplemental Table S2). The proteins identified exceeded the criterion (log2FC> 2, *P* < 0.05) and are possible Ola1p interactors and are presented in supplemental Tables S3 (10 min heat stress) and S4 (20 min heat stress).

### Experimental proof of Ola1p transport from mitochondria to LDs

**Fig. 4:**
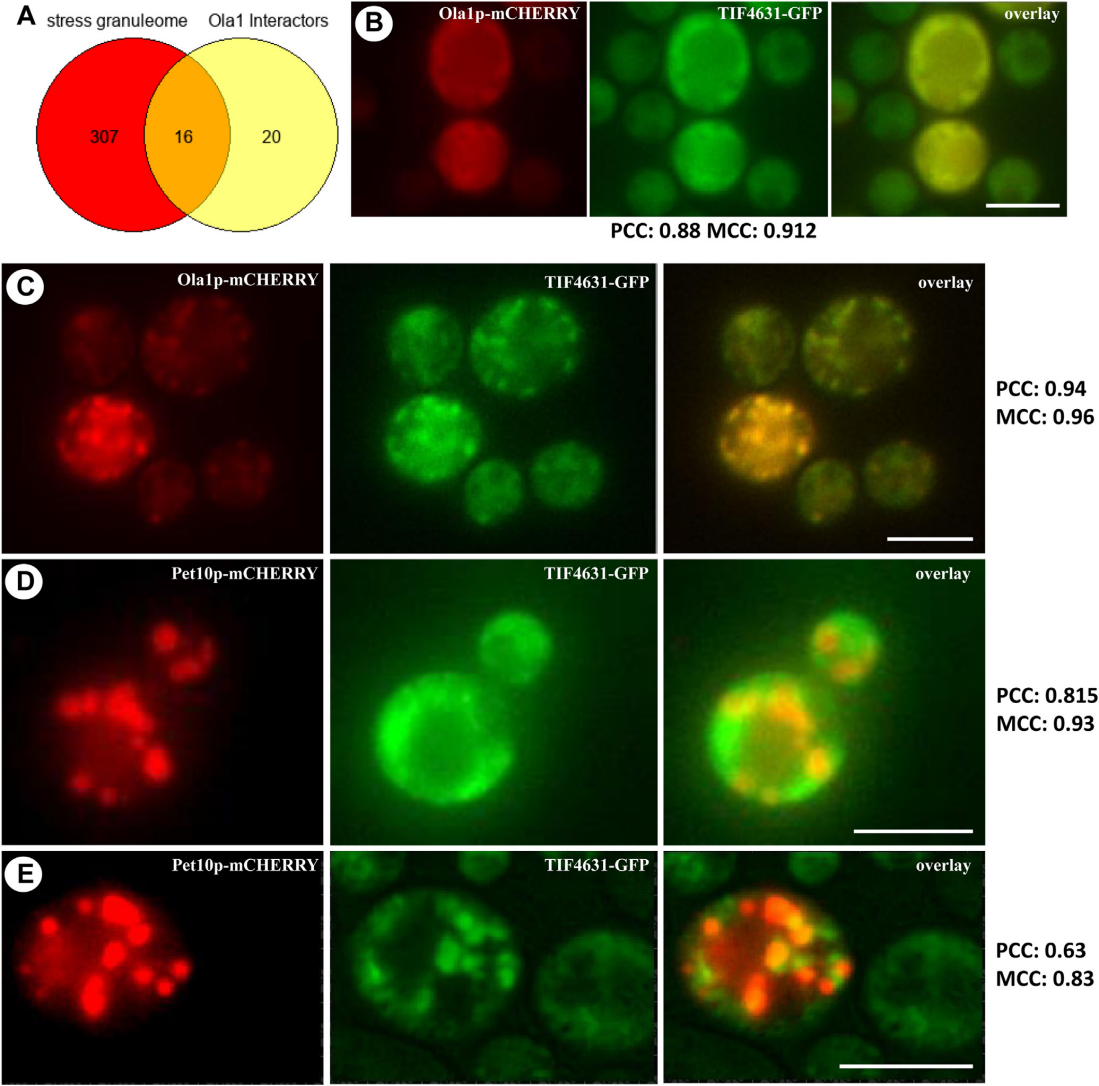
Ola1p as part of SGs. A: Venn diagram comparing Ola1p interactors identified by proximity labeling and proteins that are part of the stress granuleome. Numbers indicate identified proteins of the respective studies. Of all Ola1p interactors, only candidates were chosen that fulfil stringent criteria (log2FC> 2, *P* < 0.05). B and C: The strain BY4741 TIF4631-GFP::HIS3MX6 YEplac181-GPD-*OLA1*-mCHERRY was used. D and E: The strain BY4741 TIF4631-GFP::HIS3MX6 YEplac181-GPD-*PET10*-mCHERRY was analyzed. B and D: Unstressed cells were studied. C and E: A heat stress (20 min, 46°C) was applied. Scale bars represent 5 μm.

We discovered a high enrichment for proteins specific for the stress granuleome (32), the proteome of SGs (representation factor: 8.3; *P* < 0.0000) (Fig. 4A).

**Fig. 5:**
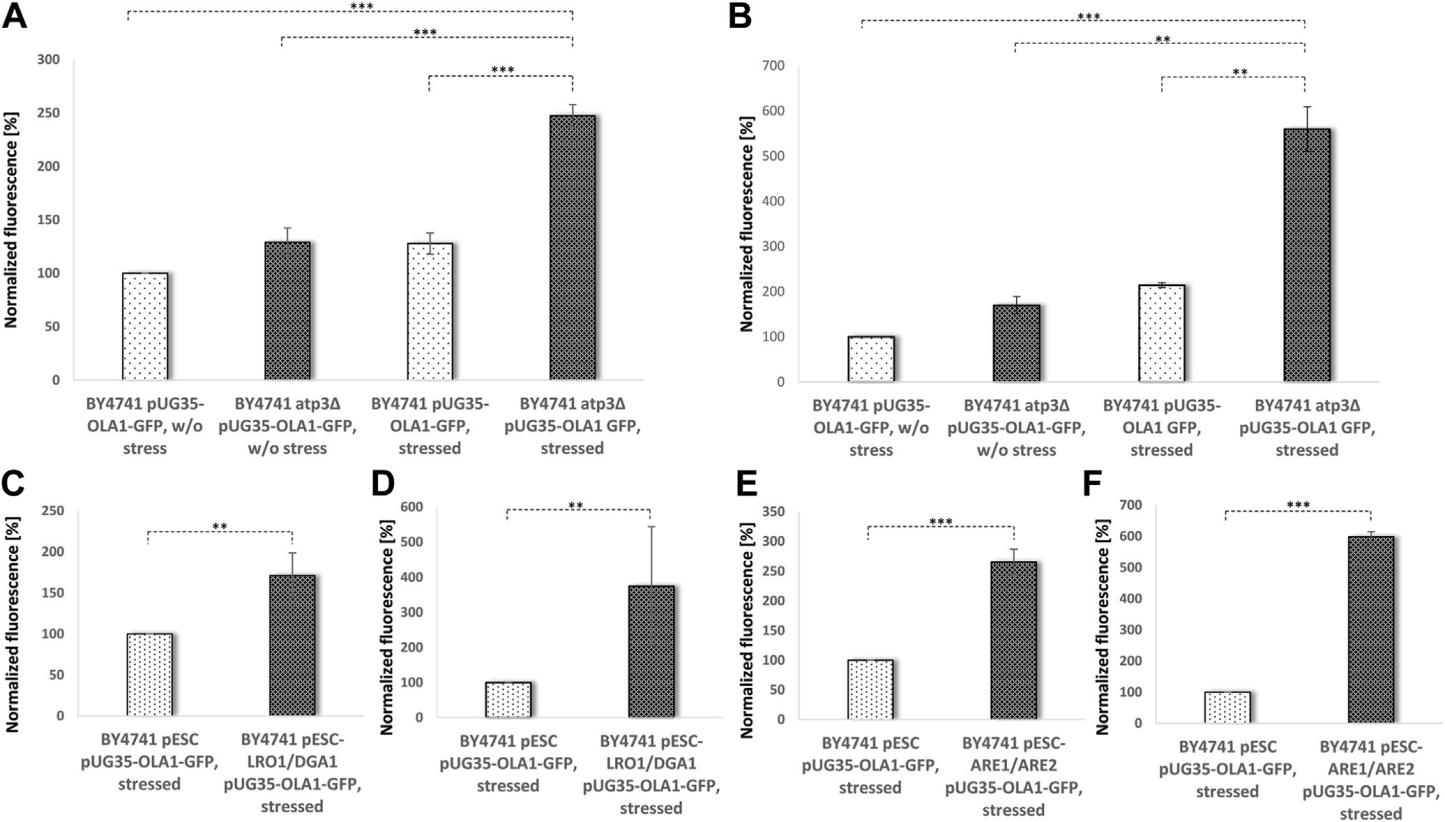
The role of mitochondrial membrane potential and LD stimulation on protein shuttling. The strains BY4741 pUG35-*OLA1*-GFP, BY4741 atp3Δ pUG35-*OLA1*-GFP, BY4741 pESC pUG35-*OLA1*-GFP, BY4741 pESC-*LRO1/DGA1* pUG35-*OLA1*-GFP, and BY4741 pESC-*ARE1/ARE2* pUG35-*OLA1*-GFP were used in these experiments. Mitochondria as well as LDs were isolated from stressed (46°C, 10 min) as well as control cells. Fluorimetric analysis of isolated mitochondria is presented in (A), (C), and (E). Fluorimetric analysis of LDs is shown in (B), (D), and (F). A and B: The loss of MMP (matrix metalloproteinase) induced by an *ATP3* deletion was analyzed. C–F: The effect on protein shuttling upon LD stimulation was evaluated. C and D: The LD levels were increased by overexpression of *LRO1* and *DGA1*. E and F: By overexpression of *ARE1* and *ARE2*. One-way ANOVA with Tukey post hoc test in (A) and (B) resulted in *P* < 0.0001. C–F: A Student's *t*-test analysis was performed.

Next, we aimed to experimentally confirm the enrichment of SG-specific proteins. We expressed both, the bona fide SG protein TIF4631 (33) and Ola1p fused to fluorescent proteins (TIF4631-GFP and Ola1p-mCHERRY). Following the application of stress (20 min at 46°C), a strong relocalization of Ola1p-mCHERRY and TIF4631-GFP was seen (Fig. 4B, C). A PCC of 0.94 indicates a high degree of colocalization between Ola1p-mCHERRY and TIF4631-GFP. This is consistent with a previous publication (26), which reported the presence of Ola1p in SGs. Furthermore, certain TIF4631-GFP-labeled SGs clearly attach to or reside on LDs (Fig. 4E). A PCC of 0.63 indicates that most, but not all, TIF4631-GFP-stained granules are near LDs. Previously, it was shown that mitochondrial import of misfolded proteins for LON protease degradation depends strictly on mitochondrial membrane potential. The uncoupler CCCP totally inhibited the transport of such proteins into the mitochondrial matrix (17). As a result, we decided to investigate the transfer of Ola1p to mitochondria or LDs in mutant cells with mitochondrial DNA depletion and hence loss of mitochondrial membrane potential. We recently discovered that spontaneous mutations in respiratory-deficient yeast strains seem to re-establish mitochondrial membrane potential (34, 35). As a result, we chose a strain with a deletion of the ATP synthase's gamma subunit, which lacks suppressor mutations. Mitochondria and LDs were extracted from stressed (46°C for 10 min) and unstressed cells expressing Ola1p-GFP ectopically. The GFP signal was greatly enhanced in cells without mitochondrial membrane potential, especially when heat stress was applied. This indicates that Ola1p cannot be transported into the mitochondrial matrix for degradation and instead accumulates at the mitochondrial outer surface, confirming the findings of Ruan *et al.* (17). At isolated LDs (in both, control and mitochondrial membrane-deficient cells), the Ola1p-GFP fluorescence was considerably brighter (Fig. 5B). The failure of Ola1p degradation appears to initiate a transfer of this protein from mitochondria to LDs.

As shown above, the kinetics of this process is also significantly affected by the availability and presence of LDs. Overexpression of either Lro1p and Dga1p (two diacylglycerol acyltransferases) or Are1p and Are2p (two Acyl-CoA:sterol acyltransferases) increases cellular LD (11). Mitochondria and LDs were extracted from controls and both heat-stressed strains with induced LD concentration. Following LD stimulation, there was a significant accumulation of *OLA1*p-GFP at mitochondria (Fig. 5C, E) as well as LDs (Fig. 5D, F). Therefore, the LD content clearly affects the Ola1p transport mechanism from mitochondria to LDs.

**Fig. 6:**
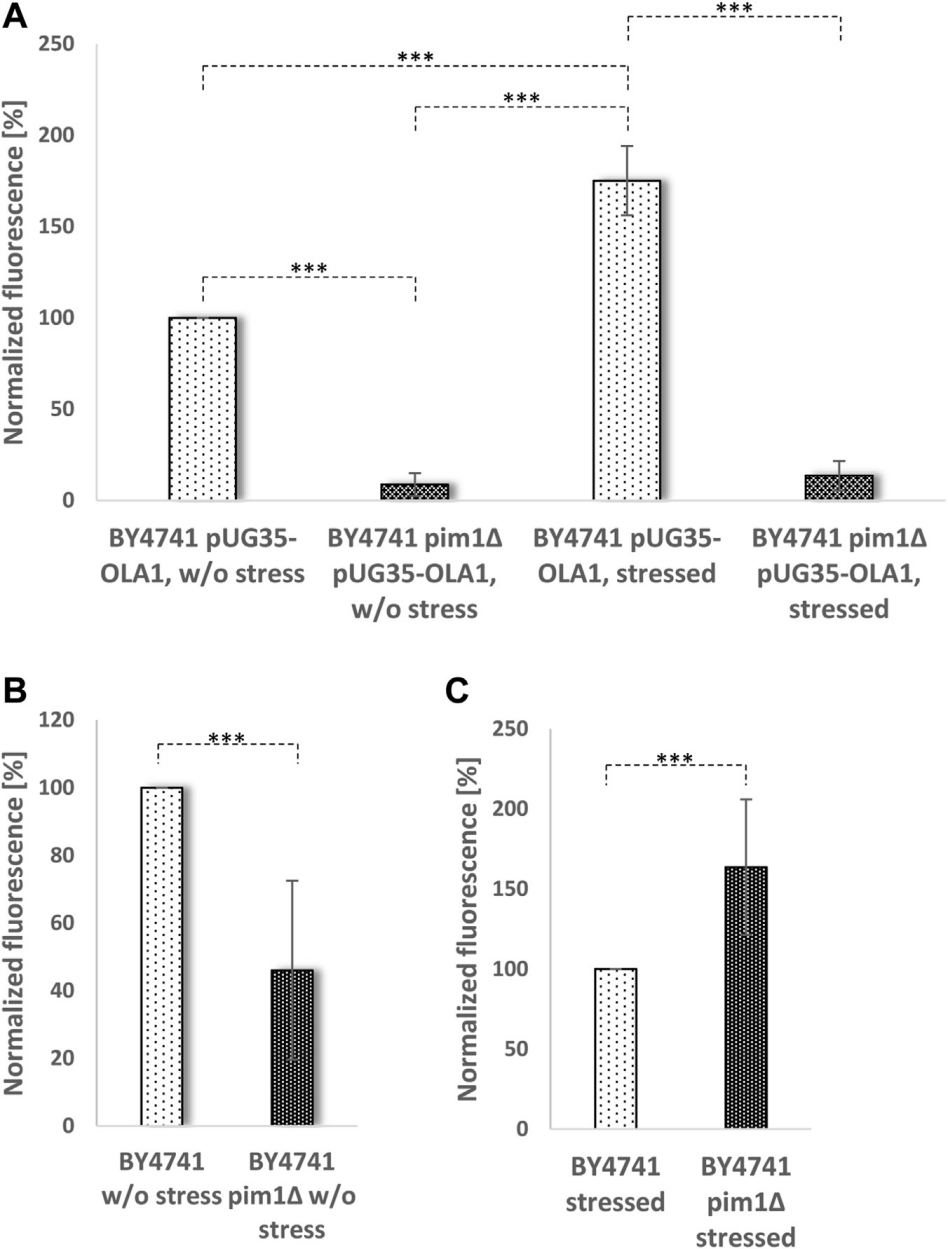
The interplay of MAGIC and LDs. The strains BY4741 pUG35-*OLA1*-GFP and BY4741 *pim1Δ* pUG35-*OLA1*-GFP were used for this study. Fluorimetric analysis of isolated mitochondria is shown in (A). B and C: Fluorimetric measurements of isolated LDs are presented. One-way ANOVA with Tukey post hoc test: *P* < 0.0001.

Furthermore, we wanted to investigate the Ola1p transport in the context of the absence of the mitochondrial matrix resident LON protease (an essential component of MAGIC). Strikingly, when this mitochondrial protease is deleted, the Ola1p-GFP concentration of mitochondria isolated from stressed and unstressed cells is drastically reduced (Fig. 6A). Ola1p was also identified in lower concentrations in isolated LDs derived from unstressed cells in the absence of the mitochondrial protease (Fig. 6B). Upon stress application, however, a considerable accumulation of Ola1p at LDs was detected, which even exceeded the quantity of Ola1p at LDs in wildtype cells (Fig. 6C). This discovery highlighted the relevance of LDs in protein homeostasis, especially in the absence of the mitochondrial LON protease.

Finally, we wanted to determine if LDs are involved in the dissolution or degradation of protein aggregates/SGs. Therefore, we subjected cells with chromosomal expression of an Ola1p-GFP construct that either contained or lacked LDs (*are1Δ are2Δ lro1Δ dga1Δ)* to heat stress (20 min at 46°C).

**Fig. 7:**
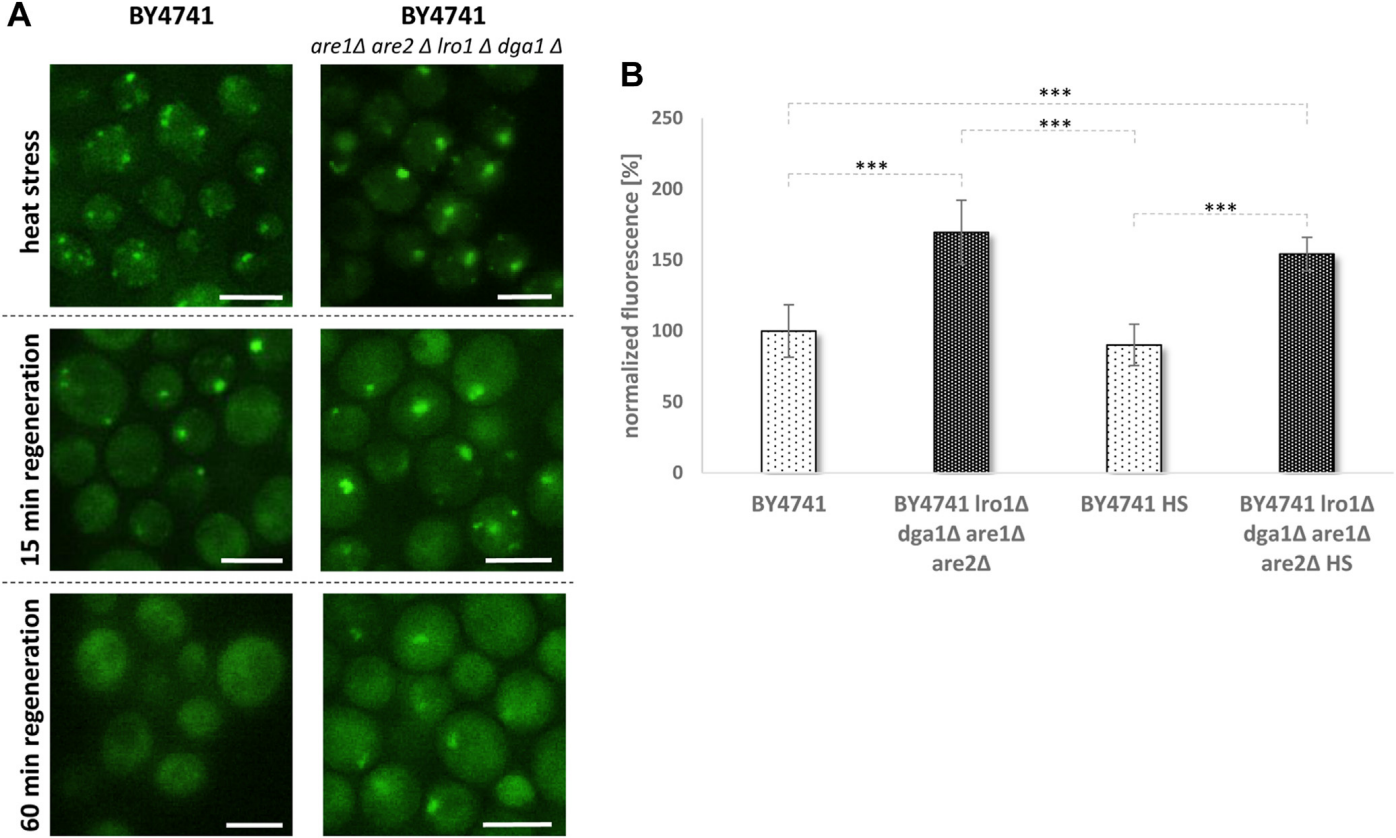
Dissolution of SGs. This study was performed with the strains BY4741 *OLA1*-GFP::hph and BY4741 *are1Δ are2Δ lro1Δ dga1Δ OLA1*-GFP::hph. A: Fluorescence microscopy images show the formation and dissolution of Ola1p-GFP aggregates. Heat stress was applied for 20 min at 46°C. Cells were regenerated in SC medium at 28°C. B: The amount of Ola1p-GFP was quantified in unstressed as well as stressed cells (20 min at 46°C; 15 min regeneration) using a fluorescent plate reader. One-way ANOVA with Tukey post hoc test: *P* < 0.01. Scale bars represent 5 μm.

Following stress treatment, 100% of cells from both strains formed some sort of SGs/protein aggregates (Fig. 7A). The SGs were substantially higher in number in LD-containing cells. SGs began to dissolve with a similar rate 15 min after the heat shock ended (LD-containing cells: 80% of SGs; LD-lacking cells: 78% of SGs). However, the dissolution of SGs/protein aggregates became more efficient in LD-containing cells over time. After 60 min of regeneration, just 8% of cells still retained SGs, whereas 47% of cells in the LD-deficient strain had aggregates (Fig. 7; *P* < 0.01). After 90 min, approximately 25% of LD-deficient cells were still unable to eliminate the aggregates. Notably, in both backgrounds, a subpopulation of cells (less than 10% of all cells) failed to properly remove the aggregates. Ola1-GFP aggregates were clearly detectable in these cells even after 2.5 h (data not shown). We used fluorometric measurements to quantify the decreased capacity of LD-deficient cells to eliminate SGs/aggregates from cytosol (Fig. 7B).

## Discussion

SGs are membrane-less cytoplasmatic aggregates formed in response to stress (e.g., heat shock and oxidative stress). The composition of these SGs is very dynamic, changing dramatically depending on the stress and growth phases. Translationally arrested mRNAs, the 40S ribosomal subunit, the 48S initiation complex, several translation initiation factors, and polyA-binding proteins were identified as SG components (36, 37, 38). The fundamental function of SGs is yet unknown, but they appear to be vital in continuing growth and translation after stressful conditions have passed (38, 39). Their disintegration is just as important as their formation. SG dissolution appears to be a highly regulated process involving “disassembly engaged proteins,” chaperones, and SUMOylation pathways (37, 38, 40). Failure to eliminate SG results in the formation of either persistent aggregates or abnormal aggregates, which eventually lead to neurodegenerative disorders (37, 38). Recent evidence suggests that SGs are closely associated with mitochondria in both mammalian (16) and yeast cells (25, 38). Because two SG components, Ola1p and Mmi1p, are MAGIC targets in yeast cells (17), it appears plausible that mitochondria are involved in SG dissolution. Furthermore, we noticed a significant association between SGs and LDs. The link between SG and LDs and mitochondria appears to exist in higher cells as well, as a triade of LDs, SGs, and mitochondria was observed in human human embryonic kidney 293 cells (16). Indeed, we were able to demonstrate that SGs (or at least protein components that are part of SGs) are shuttled to mitochondria before being passed on to LDs in yeast cells. We were able to show that this attachment to LDs may act as a type of detoxifying process, as demonstrated by Ola1p.

So far, our understanding of Ola1p in *S. cerevisiae* is limited. Ola1p, a P-loop ATPase (41) is highly stress responsive (42) and expressed in response to either DNA replication stress or hydrogen peroxide treatment. Ola1p has been shown to interact with the 26S proteasome, to be involved in protein translation control (43) and to help stabilize misfolded proteins (44). LDs aid to suppress the quantity of Ola1p, especially when cellular stress is applied. From the three models of LD-dependent protein homeostasis put forward in the introduction (first: ubiquitination at the ER and elimination via microlipophagy; second: dissolution of inclusion bodies via sterol esters; and third: redirection of damaged proteins from mitochondria to LDs), it seems that the last case seems to be the most correct, with the LDs assisting the overwhelmed MAGIC pathway.

Ola1p protein content is more than 1.5-fold higher in yeast cells devoid of LDs. The presence of Ola1p appears to be beneficial to cells during normal growth. In contrast, high levels of Ola1p seem to be less favorable during periods of stress linked with the translocation of Ola1p into SGs. These results were consistent with findings in human cell lines, where *OLA1* was identified as a suppressor of the antioxidant stress response (45). Using the LD and SG resident “super aggregator” Ola1p as an example of a protein that can be sequestered from SGs and mitochondria to LDs to potentially ameliorate proteotoxic stress, we highlight the complex relationship between LDs, SGs, and mitochondria under heat stress. In addition to the purely scientific questions that were addressed in this work, an in vitro reporter was also established that makes it possible to study the association of proteins or protein aggregates with LDs. In addition, it would also be exciting to use this reporter for aggregate dissolution studies in the future.

## Data Availability

All data are contained within the article and supplemental data.

## Supplemental data

This article contains supplemental data.

## Conflict of interest

The authors declare that they have no conflicts of interest with the contents of this article.

## Figures and Tables

**Supporting Figure S1:**
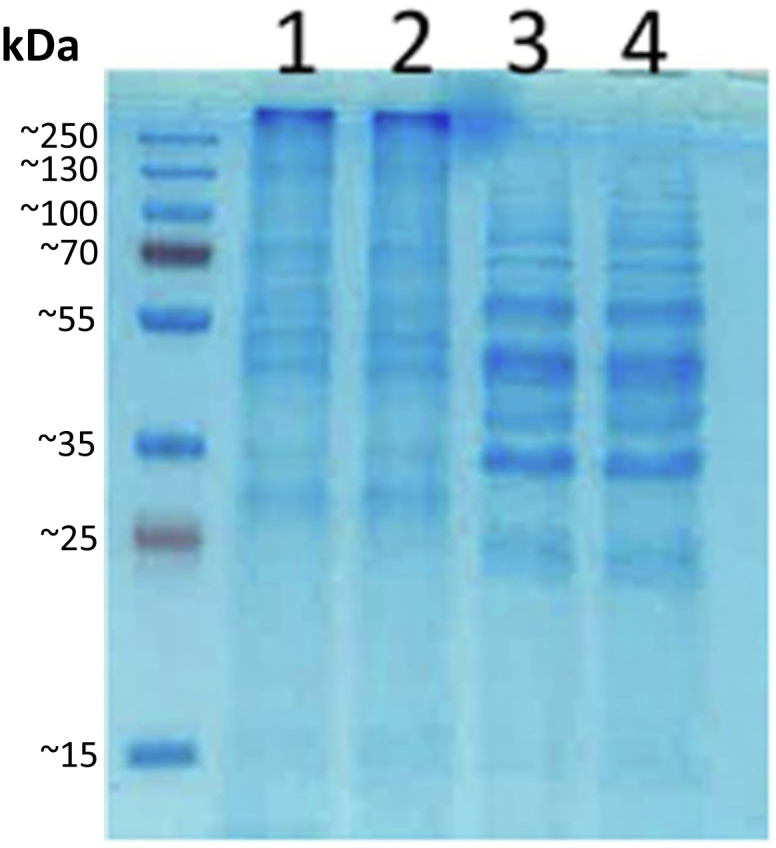
Loading control for Fig. 2E. *1*) Protein extracts from nonapoptotic mitochondria; *2*) Protein extracts from apoptotic mitochondria; *3*) LD extracts isolated from nonapoptotic cells; and *4*) LD extracts isolated from apoptotic cells.

## Supplemental Data

Supporting Table S4

Supporting Movie This video is a 3D stack of BY4741 pUG34-Ola1-GFP YEplac181-GPD-PET10-mCHERRY cells (see Fig. 2D). It demonstrates that some Ola1p containing aggregates attach to LDs (uploaded file).
