# Supplementary material for: Ola1p trafficking indicates an interaction network between mitochondria, lipid droplets, and stress granules in times of stress
Source: J Lipid Res. 2023 Nov 9;64(12):100473. doi: 10.1016/j.jlr.2023.100473 (PMC10757043; doi:10.1016/j.jlr.2023.100473)
Supplement: Supporting Table S3 [file mmc3.docx]

| **Gene** | **foldchange (log2FC)** | **p-value** |
| --- | --- | --- |
| TRR1 | 7,2 | 1,94E-04 |
| YHB1 | 3,6 | 3,39E-03 |
| CAF20 | -3,1 | 5,82E-03 |
| RVS161 | 3 | 9,29E-03 |
| CDC21 | 3,5 | 1,23E-02 |
| RPL16A | 2,6 | 1,70E-02 |
| CPR1 | 2,7 | 1,82E-02 |
| VPS5 | 2,4 | 1,87E-02 |
| YIL108W | 3,3 | 2,24E-02 |
| LIA1 | 5,1 | 2,75E-02 |
| TOM70 | 3,9 | 2,83E-02 |
| GDH1 | 3 | 2,96E-02 |
| ROD1 | 2,6 | 3,00E-02 |
| BAT2 | 3,3 | 3,09E-02 |
| APA1 | 4,6 | 3,13E-02 |
| IMD3 | 4,6 | 3,92E-02 |
| TMA19 | 2,7 | 4,08E-02 |
| YER134C | -2 | 4,68E-02 |
