# Supplementary material for: Ola1p trafficking indicates an interaction network between mitochondria, lipid droplets, and stress granules in times of stress
Source: J Lipid Res. 2023 Nov 9;64(12):100473. doi: 10.1016/j.jlr.2023.100473 (PMC10757043; doi:10.1016/j.jlr.2023.100473)
Supplement: Supporting Table S4 [file mmc4.docx]

| **Gene** | **foldchange (log2FC)** | **p-value** |
| --- | --- | --- |
| TRR1 | 8,4 | 1,73E-05 |
| CPR1 | 4,5 | 1,17E-03 |
| YHB1 | 4 | 1,95E-03 |
| LIA1 | 6,5 | 2,08E-03 |
| GDH1 | 5,6 | 2,84E-03 |
| RVS161 | 3,7 | 3,89E-03 |
| CDC21 | 3,9 | 5,78E-03 |
| PAB1 | 4 | 8,97E-03 |
| TMA19 | 3,7 | 1,01E-02 |
| PUB1 | 3,8 | 1,06E-02 |
| RCK2 | 3,8 | 1,12E-02 |
| SEC53 | 4,1 | 1,33E-02 |
| ENO1 | 4,1 | 1,38E-02 |
| APA1 | 5,3 | 1,48E-02 |
| TOM70 | 4,4 | 1,48E-02 |
| ROD1 | 3,4 | 1,69E-02 |
| RSC8 | 2,6 | 2,10E-02 |
| ALD4 | 4,1 | 2,50E-02 |
| GLN1 | 3,6 | 2,75E-02 |
| BAT2 | 3,5 | 3,32E-02 |
| ENO2 | 3,3 | 3,58E-02 |
| GLN4 | 3,4 | 3,79E-02 |
| SSE1 | 2,2 | 4,00E-02 |
| FAA4 | 2,1 | 4,27E-02 |
| ALA1 | 2,6 | 4,37E-02 |
| MCM4 | 2,9 | 4,46E-02 |
| TIF5 | 2,2 | 4,68E-02 |
| UBC4 | 2,5 | 4,73E-02 |
| VTC2 | 3,1 | 4,84E-02 |
